# Predictive risk score for unplanned 30-day rehospitalizations in the French universal health care system based on a medico-administrative database

**DOI:** 10.1371/journal.pone.0210714

**Published:** 2019-03-12

**Authors:** Vanessa Pauly, Hélène Mendizabal, Stéphanie Gentile, Pascal Auquier, Laurent Boyer

**Affiliations:** 1 Aix-Marseille University, Public Health, Chronic Diseases and Quality of Life—Research Unit, La Timone Medical University, Boulevard Jean-Moulin, Marseille, France; 2 Service d’Information Médicale, Public Health Department, La Conception Hospital, Assistance Publique—Hôpitaux de Marseille, Marseille, France; 3 Cellule Évaluation Médicale, Public Health Department, La Conception Hospital, Assistance Publique—Hôpitaux de Marseille, Marseille, France; Duke-NUS Medical School, SINGAPORE

## Abstract

**Background:**

Reducing unplanned rehospitalizations is one of the priorities of health care policies in France and other Western countries. An easy-to-use algorithm for identifying patients at higher risk of rehospitalizations would help clinicians prioritize actions and care concerning discharge transitions. Our objective was to develop a predictive unplanned 30-day all-cause rehospitalization risk score based on the French hospital medico-administrative database.

**Methods:**

This was a retrospective cohort study of all 2015 discharges from acute-care inpatient hospitalizations in a tertiary-care university center comprising four hospitals. The study endpoint was unplanned 30-day all-cause rehospitalization via emergency departments, and we collected sociodemographic, clinical, and hospital characteristics based on hospitalization database computed for reimbursement of fees. We derived a predictive rehospitalization risk score using a split-sample design and multivariate logistic regression, and we compared the discriminative properties with the LACE index risk-score.

**Result:**

Our analysis included 118,650 hospitalizations, of which 4,127 (3.5%) led to rehospitalizations via emergency departments. Variables independently associated with rehospitalization were age, gender, state-funded medical assistance, as well as disease category and severity, Charlson comorbidity index, hospitalization via emergency departments, length of stay (LOS), and previous hospitalizations 6 months before. The predictive rehospitalization risk score yielded satisfactory discriminant properties (C statistic: 0.74) exceeding the LACE index (0.66).

**Conclusion:**

Our findings indicate that the possibility of unplanned rehospitalization remains high for some patient characteristics, indicating that targeted interventions could be beneficial for patients at the greatest risk. We developed an easy-to-use predictive rehospitalization risk-score of unplanned 30-day all-cause rehospitalizations with satisfactory discriminant properties. Future works should, however, explore if other data from electronic medical records and other databases could improve the accuracy of our predictive rehospitalization risk score based on medico-administrative data.

## Background

Reducing 30-day rehospitalizations is a priority of health care policies in Western countries [1,2]. Unplanned rehospitalizations are common [3,4] and costly [3,5], reflecting poor quality inpatient care [6–8] and poorly coordinated transitions between hospitals and homes [9]. Despite the growing literature on this issue, unplanned rehospitalizations are still poorly understood and controlled [4]. We need to better understand its determinants and be able to identify patients at high risk of rehospitalization in order to improve quality of care and reduce rehospitalizations and associated health care costs [10]. To date, the majority of studies have focused on particular conditions, *e*.*g*., patients with specific diagnoses [11–14] or socio-demographic characteristics like old age [4,15,16], limiting their generalizability [17,18]. Despite the need to target patients at high risk of rehospitalizations in order to propose cost-effective interventions at hospital level [3,19], there is very limited research addressing all-cause unplanned rehospitalizations.

In addition, most of the studies focused on this issue were performed in the United States of America (USA) [20] where the influence of access to care and health insurance [21] on unplanned rehospitalization proves to be a complex factor to control, leading to paradoxical and contradictory findings [22–25]. Exploring all-cause unplanned rehospitalizations in different national health care systems may thus provide new information [4,26], and the French Health System (FHS) could possibly offer an interesting approach given the opportunity to better control this factor as French universal health coverage guarantees access to the most appropriate care regardless of cost [27]. Briefly, the FHS combines universal coverage with a public–private mix of hospital and ambulatory care, offering a higher volume of service provision than the USA [27]. Complementary insurance needs to be contracted, leading to out-of-pocket expenses, but the FHS coverage increases when a patient’s costs increase, without deductibles. The majority of patients with severe illnesses are exempted from complementary insurance [28]. Finally, policymakers in France have not implemented a pay-for-performance system based on rehospitalization indicators, yet hospitals have recently been put under pressure due to the public diffusion of rehospitalization rates, considered as comparative quality-of-care indicators between institutions [29]. To the best of our knowledge, only a few studies have been carried out in the FHS [4], and no study has yet to specifically examine the determinants of all-cause rehospitalizations in France.

Our study objective was to develop a predictive unplanned 30-day all-cause rehospitalization risk score based on the French hospital medico-administrative database.

## Methods

### Study design

This was a retrospective cohort study of all acute-care inpatient hospitalization cases discharged from January 1 to December 31, 2015, from the largest university health center in south France (*Assistance Publique–Hôpitaux de Marseille*, APHM). All data were collected from the APHM Hospital database computed for reimbursement of fees including both the PMSI (PMSI—*Programme de Médicalisation des Systèmes d’Information*) database as well as administrative data. The PMSI is the French medico-administrative database for all hospitalizations based on diagnosis related-groups (DRG) that we can group into significant diagnostic categories.

### Study setting and inclusion criteria

The APHM is public tertiary-care center comprising four hospitals (La Timone, La Conception, Sainte-Marguerite, and North), 3,400 beds, and 2,000 physicians. Approximately 300,000 hospitalizations are recorded every year at the APHM, involving approximately 210,000 patients. All acute-care hospitalizations were included in this study. We excluded hospitalizations in ambulatory care unit (*i*.*e*., ambulatory surgery, radiotherapy, dialysis, chemotherapy, transfusions) as well as in-hospital mortalities.

### Study outcome

The study outcome was unplanned 30-day all-cause rehospitalization, defined as any cause of admission via emergency departments in any acute care wards within 30 days of discharge. To calculate this outcome, the unique and individual identifying variable was used to track rehospitalizations 30 days following discharge. We excluded patients with identification problems in the database. No more than one rehospitalization for each discharge was taken into account. Readmission via the emergency department was employed to identify unplanned rehospitalizations [30].

### Collected data

The following data were collected from the PMSI:

- socio-demographic characteristics: age, gender, state-funded medical assistance (Aide Médicale d’Etat, AME) (*i*.*e*., health cover for undocumented migrants), and free universal health care (Couverture Maladie Universelle, CMU) (*i*.*e*., universal health coverage for those not covered by employment/business-based schemes);
- clinical characteristics: category of disease based on the 10th revision of the International Statistical Classification of Diseases [31], disease severity and the Charlson comorbidity index based on the algorithm developed by Quan et al. [32]; type of hospitalization (medical, surgical or obstetrical). Disease severity (no or low severity, moderate–high severity or not determined for short hospitalizations) as well as the categories of disease are constituted from the Diagnosic Related Groups issued from the PMSI’s algorithm which takes into account age and other comorbidities, medical or chirurgical procedures. This algorithm is available on the ATIH Website [33]. Categories of disease are clusters of distinct DRG. This algorithm is used for all the French hospitals (private, public and university ones) and is reproducible.
- the LACE index: a widely used instrument for predicting the risk of unplanned rehospitalization within 30 days of discharge [34,35]. It is computed from four variables: length of stay (LOS), admission via emergency departments, Charlson comorbidity index [32], and previous admission via emergency departments six months before. Scores range from 0, indicating the lowest risk, to 19, indicating the highest risk;
- Hospitalization characteristics: patient origin (home or other hospital institution), hospitalization via emergency departments, LOS, destination after hospital discharge (home or transfer to other hospital institution), hospitalization via emergency departments 6 months before.

### Statistical analyses

The unit of analysis was the hospitalization. Descriptive analyses for the socio-demographic, clinical, and hospitalization data were expressed as frequencies and percentages. Chi-squared tests were employed to compare socio-demographic, clinical, and hospitalization data between unplanned 30-day all-cause rehospitalized and non-rehospitalized patients. Multivariate logistic regression was then performed to identify variables potentially associated with unplanned 30-day all-cause rehospitalization, after adjusting for confounding factors. Variables relevant to the model were selected based on a threshold p-value (≤0.2) in the univariate analysis and had to be non collinear with other variables introduced in the model. Odds ratios (OR) with confidence intervals (CI) were calculated.

Based on the beta coefficients issued from the multivariate logistic regression, we developed a 0-to-100 point score for rehospitalization risk prediction using a regression-coefficient-based scoring method [36,37]. The number of points assigned to each modality equaled its regression (beta) coefficient multiplied by 100 and divided by the highest score of rehospitalization (corresponding to the sum of the highest beta coefficient of each variable). We then calculated each patient’s final score by totaling their points. The area under the receiver operating characteristics curve (AUC under ROC) was derived to evaluate this risk score’s capacity of discriminating between rehospitalized and non-rehospitalized patients. The AUC ranges from 0.5 to 0.99, with higher values signifying higher model discrimination. The AUC of the predictive rehospitalization risk-score was then compared to the AUC of the LACE index score [38], computed on the same database. To compare these two AUCs, we used Chi-square statistic developed by Gönen [39].

In addition to the AUC metric, we used other metrics based on a threshold value determined as the best trade-off between sensitivity and specificity. For this cutoff, we provide the following metrics for each score (*i*.*e*., the predictive rehospitalization risk-score and the LACE index): sensitivity, specificity, the Youden Index, the accuracy and the F1 score:

$$Sensitivity=\frac{true\;positive}{true\;positive+false\;negative}$$

$$Specificity=\frac{true\;negative}{true\;negative+false\;positive}$$

Youdenindexscore=sensitivity+specificity−1

$$Accuracy=\frac{true\;positive+true\;negative}{N}$$

$$Precision=\frac{true\;positive}{true\;positive+false\;positive}$$

$$F1\;score=2*\frac{sensitivity*precision}{sensitivity+precision}$$

Then, we assessed the robustness of the predictive rehospitalization risk score. Following the methodology proposed by Tuffery [37], the data were split into two samples: a training sample including 2/3 of the data and a test sample including the remaining 1/3. Using the training datasets, risk scores were built for each independently-associated factors of 30-day all-cause rehospitalization, previously determined and using the multivariate logistic regression. The AUCs obtained for the training and testing datasets were then compared. The model was considered robust if the AUCs between the testing and training datasets were similar.

Lastly, we computed the 30-day rehospitalization rates to each class (10 by classes) of the predictive risk score and test the association using the Chi-square test.

Statistical significance was defined as p <0.05. The statistical analyses were performed with Statistical Analysis Software (SAS), Version 9.4 (SAS Institute).

### Ethics and consent to participate

The datasets generated and/or analyzed during the current study are issued from the Assistance Publique des Hôpitaux de Marseille (APHM) hospitalization database computed for reimbursement of fees. Patients are informed by the hospital that their data may be analyzed for research purpose, consequently respecting the French law for research that does not require explicit or written consent of the patient. No additional data out of this database has been computed for the study. The use of such database is governed by a local ethic committee (named CIL-APHM) and declared under the following authorization numbers 1305855 for medical data and 2012–1 for administrative ones. According to the French law, there is no need to ask to another relevant ethical review board or relevant regulatory body.

## Results

### Rates of unplanned 30-day all-cause rehospitalization

A total of 289,358 hospitalizations (112,662 patients) were recorded in 2015 in this French University Hospital. After excluding mortalities and hospitalization in ambulatory hospitalizations care unit (ambulatory surgery, radiotherapy, dialysis, chemotherapy, transfusions), 118,650 hospitalizations (82,862 distinct patients) were included. The most common diseases were digestive disease, nervous system conditions, and cardiovascular and pulmonological diseases. We excluded 4 patients with identification problems in the database. In total, 4,127 (3,294 distinct patients) hospitalizations resulted in rehospitalizations via emergency departments 30 days after discharge (30-day re-hospitalization rate equal to 3.5%). Thirty–days rehospitalization rates according to socio-demographic, clinical, and hospitalization characteristics are presented in Table 1.

**Table 1:** Sample characteristics and rates of unplanned 30-day all-cause rehospitalizations.

	30-day re-hospitalization rates*		p-value	All (n = 118 650)	
**Sociodemographic characteristics**					
**Age**	**N (4 127)**	**%**	**<0.0001**	**N**	**% of all**
> = 75 years old	1 201	5.6		21 651	18.3
> = 18 and <75 years old	2 156	2.9		75 063	63.3
> = 1 and < = 17 years old	363	2.9		12 596	10.6
<1 year	407	4.4	** **	9 340	7.9
**Gender**	** **	** **	**<0.0001**	** **	** **
Male	2 377	4.0		59 874	50.5
Female	1 750	3.0	** **	58 776	49.5
**State-funded medical assistance**			**<0.0001**		
Yes	142	7.4		1 921	1.6
No	3 985	3.4		116 729	98.4
**Free universal health care**	** **	** **	**<0.0001**	** **	** **
Yes	600	4.7		12 894	10.9
No	3 527	3.3		105 756	89.1
**Clinical characteristics**					
**Category of disease**	** **	** **	**<0.0001**	** **	** **
Digestive	600	4.9		12 159	10.3
Orthopedic—Trauma	218	2.7		8 000	6.7
Multiple or complex trauma	11	3.8		288	0.2
Rheumatology	81	2.2		3 656	3.1
Nervous systems	428	3.6		12 030	10.1
Vascular catheterization	160	2.4		6 598	5.6
Cardiovascular	340	3.7		9 167	7.7
Pulmonology	604	6.6		9 108	7.7
Ear Nose and Throat—Stomatology	80	1.6		4 903	4.1
Ophthalmology	13	0.8		1 579	1.3
Gynecology-Breast	45	2.0		2 231	1.9
Obstetrical	98	1.3		7 360	6.2
Newborns and perinatal diseases	224	3.8		5 827	4.9
Uro-nephrology and reproductive organ	274	4.1		6 754	5.7
Hematology	148	4.9		3 014	2.5
Chemotherapy—radiotherapy	155	2.0		7 852	6.6
Infectious diseases	76	5.3		1 444	1.2
Endocrinology	151	3.1		4 856	4.1
Cutaneous and subcutaneous	67	2.1		3 146	2.7
Psychiatry	83	6.6		1 264	1.1
Toxicology—Intoxication—Alcohol	96	6.0		1 611	1.4
Chronic pain palliative care	20	5.7		351	0.3
Organ Transplant	3	1.3		240	0.2
Interdisplinary activities and follow-up of patients	149	3.1		4 825	4.1
Burns	3	0.8		387	0.3
**Severity**			**<0.0001**		
No or low severe	2 121	2.7		78 014	65.8
Moderate—high severe	1 180	6.2		19 191	16.2
No determined (short stay)	826	3.9		21 445	18.1
**Charlson Comorbidity Index**			**<0.0001**		
0	2 513	3.0		84 364	71.1
1	582	4.6		12 786	10.8
2	416	4.0		10 356	8.7
3	191	5.0		3 802	3.2
4 and higher	425	5.8		7 342	6.2
**The LACE SCORE**			**<0.0001**		
Low risk	1 946	2.4	** **	81 742	68.9
Moderate risk	1 615	5.1		31 414	26.5
High risk	566	10.3		5 494	4.6
**Type of hospital stay**	** **	** **	**<0.0001**	** **	** **
Chirurgical	922	2.6		35 480	29.9
Medical	3 107	4.1		75 810	63.9
Obstetrical	98	1.3		7 360	6.2
** Hospital stay characteristics**					
**Origin of patient**	** **	** **	**0.25**	** **	** **
From home	3 878	3.5		111 973	94.4
Other (other hospital-institution)	249	3.7		6 677	5.6
**Hospitalization via emergency departments**		** **	**<0.0001**	** **	** **
No	1 06	2.2		71 846	60.5
Yes	2 521	5.4		46 804	39.5
**Length of hospital stay**	** **	** **	**<0.0001**	** **	** **
One day	265	4.4		5 979	5.0
From 2 to 3 days	1 201	2.9		42 083	35.5
From 4 to 8 days	1 417	3.1		46 251	39.0
9 and higher	1 244	5.1		24 337	20.5
**Destination on discharge**	** **	** **	**<0.0001**	** **	** **
Return to home	3 425	3.4		101 859	85.9
Other (other hospital-institution)	702	4.2		16 791	14.1
**Hospitalization via emergency departments 6 months before**			**<0.0001**		
No hospitalization	1 935	2.6		74 452	62.8
At least one previous hospitalization but not via emergency departments	719	2.5		28 591	24.1
At least one previous hospitalization via emergency departments 6 months before	1 473	9.4		15 607	13.2

* 30-days rehospitalization rate corresponds to the percentage of patients rehospitalized within 30 days for each modality of investigated factors, e.g., 3% of women have been rehospitalized within 30-days after discharge vs 4% for men.

### Factors associated with rehospitalizations

The univariate and multivariate analyses results are presented in Tables 1 and 2.

**Table 2:** Factors associated with unplanned 30-day all-cause rehospitalizations and predictive rehospitalization risk score: Multivariate analysis (n hospital stays = 118 650).

		Multivariate OR [95%CI]			Multivariate p value	Coefficient Score / Points
**Sociodemographic characteristics**						
Age					< .0001	
	> = 75 years old	1.40	1.29	1.51	< .0001	5
	[1; 74] year old	1				0
	<1 year old	1.06	0.90	1.25	0.50	1
Gender	Male	1.21	1.13	1.30	< .0001	3
	Female	1				0
State-funded medical assistance	Yes	2.14	1.78	2.58	< .0001	12
	No	1				0
**Clinical characteristics**						
Category of disease					< .0001	
	Digestive	4.58	1.47	14.35	0.0089	24
	Orthopedic—Trauma	3.17	1.01	10.00	0.0483	19
	Multiple or complex trauma	3.26	0.90	11.86	0.0723	19
	Rheumatology	2.59	0.81	8.25	0.1078	15
	Nervous systems	3.71	1.18	11.62	0.0245	21
	Vascular catheterization	3.22	1.02	10.18	0.0459	19
	Cardiovascular	3.45	1.1	10.84	0.0337	20
	Pulmonology	5.15	1.65	16.13	0.0049	26
	Ear Nose and Throat—Stomatology	2.37	0.74	7.55	0.1450	14
	Ophthalmology	1.26	0.36	4.45	0.7196	4
	Gynecology-Breast	3.78	1.17	12.28	0.0267	21
	Obstetric	1.23	0.39	3.92	0.7227	3
	Newborns and perinatal diseases	5.51	1.74	17.50	0.0038	27
	Uro-nephrology and reproductive organ	4.04	1.29	12.71	0.0168	22
	Hematology	4.42	1.4	14.00	0.0113	24
	Chemotherapy—radiotherapy	3.39	1.07	10.72	0.0375	20
	Infectious diseases	3.71	1.16	11.86	0.0272	21
	Endocrinology	3.87	1.23	12.23	0.0210	22
	Cutaneous and subcutaneous	2.24	0.70	7.16	0.1751	13
	Psychiatry	4.78	1.49	15.19	0.0085	25
	Toxicology—Intoxication—Alcohol	4.98	1.56	15.83	0.0066	26
	Chronic pain palliative care	4.36	1.27	14.90	0.0190	24
	Organ Transplant	1.21	0.24	6.06	0.8213	3
	Burns	1				0
Severity	No/ low or not determined (short stay)	1				0
	Moderate—high severe	1.37	1.25	1.50	< .0001	5
Charlson Comorbidity Index					< .0001	
	0	1				0
	1	1.14	1.03	1.26	0.0098	2
	2	1.06	0.94	1.18	0.3487	1
	3 and higher	1.29	1.17	1.43	< .0001	4
**Hospital stay characteristics**						
Hospitalization via emergency departments	Yes	2.52	2.34	2.71	< .0001	15
	No	1				0
Length of inpatient stay					< .0001	
	One day	1.26	1.10	1.44	0.0009	4
	From 2 to 8 days	1				0
	9 and higher	1.29	1.18	1.41	< .0001	4
Destination on discharge	Return to home	1.28	1.17	1.40	< .0001	4
	Other (other hospital-institution)	1				0
Hospitalization via emergency departments 6 months before					< .0001	0
	At least one previous hospitalization but not via emergency departments 6 months before	1.31	1.19	1.44	< .0001	4
	At least one previous hospitalization via emergency departments 6 months before	3.45	3.19	3.72	< .0001	20
	No hospitalization	1				0

CI: confidence interval; OR: odds ratio

Overall, the multivariate analysis confirmed the findings of the univariate analysis, except for patients who returned home being at higher risk of rehospitalization compared to those discharged to other hospitals or institutions. The following variables were found to be independently associated with rehospitalization: age, gender, state-funded medical assistance, as well as disease category and severity, Charlson comorbidity index, hospitalization via emergency departments, LOS, and previous hospitalizations 6 months before. The type of hospitalization (medical, surgical or obstetrical) was not introduced into the multivariate model due to colinearity with the disease category. The Charlson comorbidity types are provided in S1 Table.

### Development and performance of the predictive rehospitalization risk score

The scores for the predictive rehospitalization risk calculation are presented in Table 2. The characteristics accounting for the highest risks of rehospitalization were some disease categories, such as newborn and perinatal diseases (+27 points), toxicology (+26 points), pulmonology (+26 points), psychiatry (+25 points), chronic pain and palliative care (+24 points), and digestive diseases (+24 points).

Previous hospitalization via emergency departments at least 6 months before was also a crucial factor involved with being rehospitalized (+20 points), as was simply being hospitalized via emergency departments (+15 points). Concerning socio-demographic factors, patients who benefited from state-funded medical assistance were at higher risk of being rehospitalized (+12 points).

**Fig 1:**
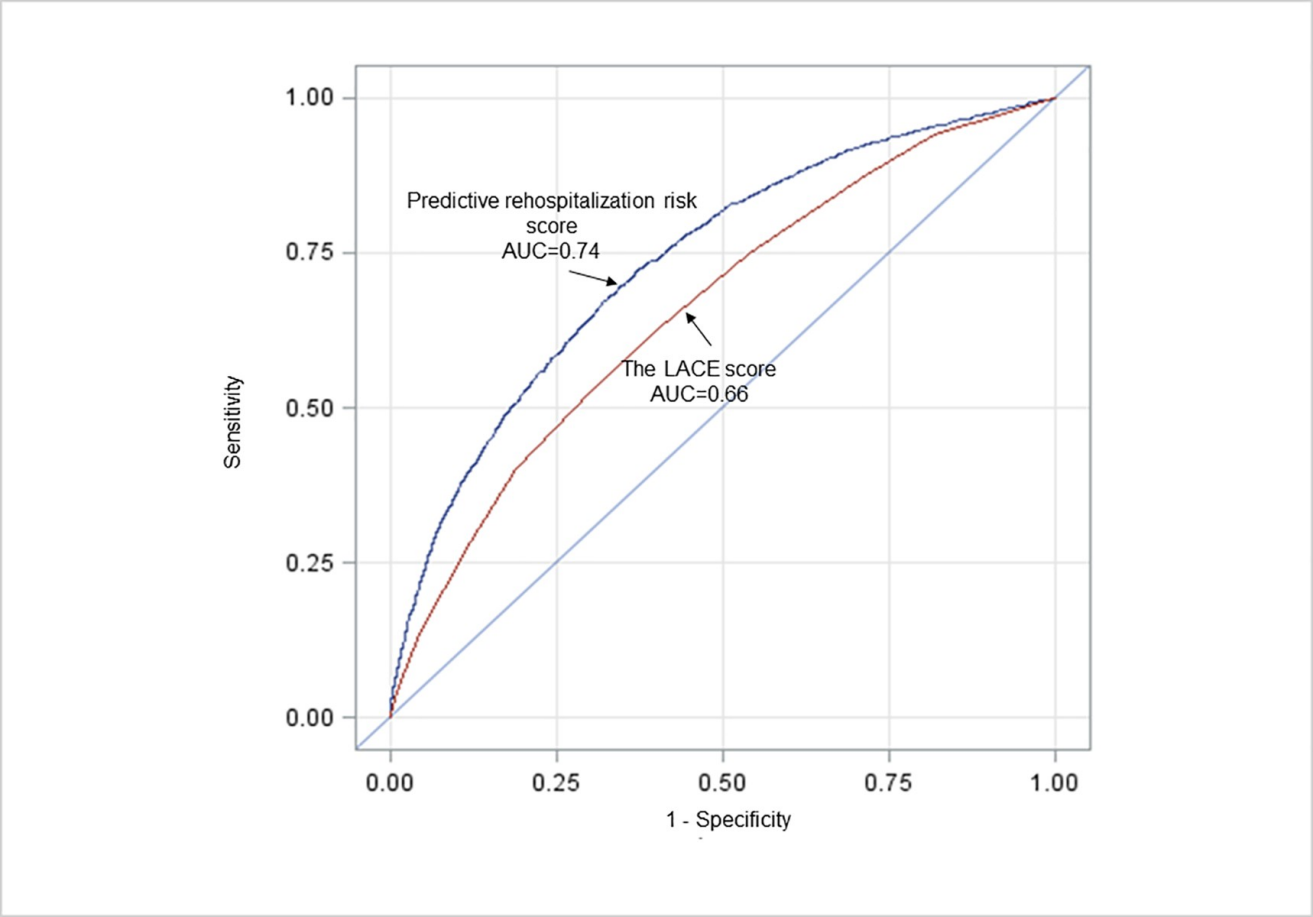
Receiver operating characteristic curves for our predictive rehospitalization risk score and LACE score (p <0.0001).

The ROC curves of the predictive rehospitalization risk score and LACE index score are presented in Fig 1. The predictive rehospitalization risk-score yielded a better AUC than that of the LACE index score (0.74 *vs*. 0.66, respectively; p <0.0001). For the rehospitalization risk score, the best trade-off between sensitivity and specificity corresponds to a probability of 0.03 (score near 42) and yield a sensitivity equal to 0.65, a specificity = 0.70, a Youden score = 0.35, an accuracy = 0.69 and a F1-score = 0.13. For the Lace score, the best trade-off between sensitivity and specificity corresponds to a probability of 0.03 (score near 6) and yield a sensitivity equal to 0.63, a specificity = 0.60, a Youden score = 0.23, an accuracy = 0.60 and a F1-score = 0.10.

**Fig 2:**
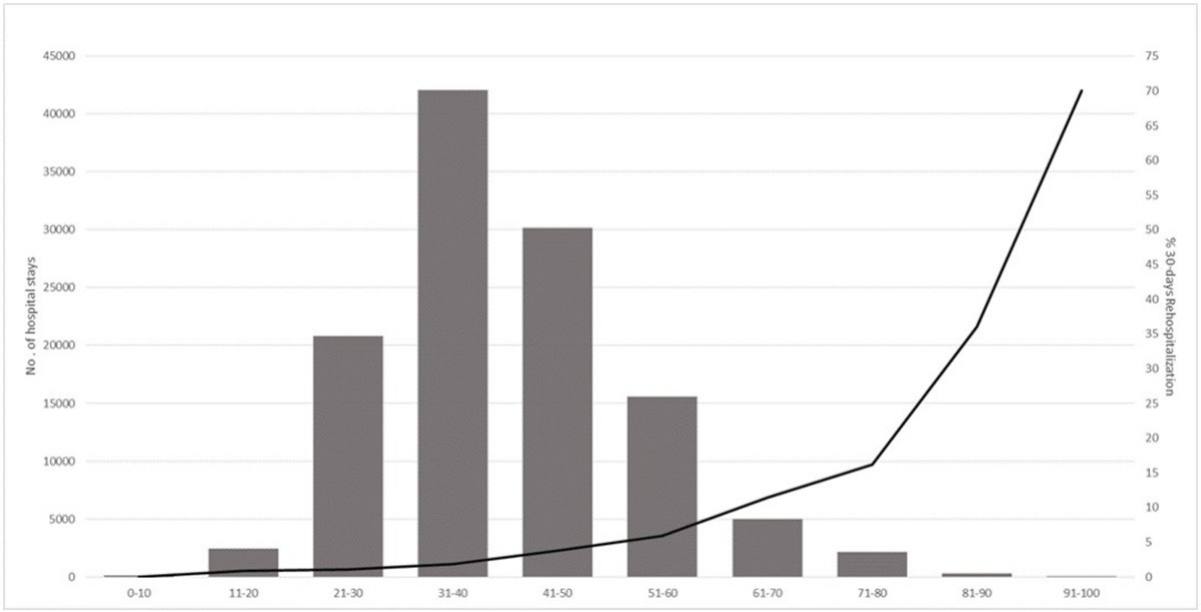
Repartition of our predictive re-hospitalization risk score (10-by-classes) and % of 30-day rehospitalization rates.

Moreover, we confirmed the accuracy of the predictive rehospitalization risk score given that 30-day rehospitalization rate increased with the predictive risk score (10- by classes), as shown in Fig 2 (p <0.0001).

The robustness of the predictive rehospitalization risk score was confirmed with similar AUCs generated for the learning (0.74) and testing (0.73) datasets.

## Discussion

The principal findings of this study can be summarized as follows. In a large sample of acute-care inpatients (82,862 patients and 118,650 hospitalizations), the rate of unplanned 30-day all-cause rehospitalization in four French university hospitals proved to be low (3.5%). Several factors predicted these rehospitalizations (*i*.*e*., age, gender, state-funded medical assistance, disease category and severity, Charlson comorbidity index, hospitalization via emergency departments, LOS, and previous hospitalization 6 months before), which could be targeted in a French national rehospitalization reduction program. Finally, we developed and internally validated an easy-to-use predictive rehospitalization risk score of unplanned 30-day all-cause rehospitalization with satisfactory discriminatory properties that can help physicians identify patients at high risk then propose adapted transitional care interventions.

The 3.5% unplanned rehospitalization rate in our study appears substantially lower than that of studies performed in other countries, even though such comparisons should be interpreted with caution due to differences in methodology (*e*.*g*., definition of unplanned rehospitalizations), and given that population studies commonly focus on particular conditions (*e*.*g*., older people, heart failure, diabetes [13,15,40]). In the few studies performed on all-cause unplanned rehospitalizations, the rates were always higher (*i*.*e*., 5.2% [41], 8.5% [19], 16.7% [42], 17.6% [3]) than in our study. We cannot exclude that patients could have been rehospitalized in another hospital and that our rate is underestimated. However, it is likely that this represents a few proportion of patients. In a study led in Switzerland, Halfon et al. [41] found that 17% of the avoidable readmissions were on a different hospital. This low unplanned 30-day all-cause rehospitalization rate may also be explained by the French universal insurance coverage since, according to Gusmano *et al*. [26], inadequate insurance coverage may result in more severe illness and consequently more hospitalizations. Such a finding has been reported in a recent study carried out in the elderly where the rehospitalization rate was 20% in the USA. *vs*. 15% in France [4]. The authors hypothesized that this discrepancy between countries was probably due to a combination of better access to primary care and longer average length of inpatient stay in France [4]. It is interesting to note that the French rehospitalization rate remains low despite a recognized lack of coordination between hospitals and primary care, in addition to a lack of preparation of discharge from the hospital in France, two factors known to be associated with rehospitalizations [16,43]. This suggests that insurance coverage may be an important factor in controlling rehospitalization that should be kept in mind in health policies in addition to more targeted interventions (*e*.*g*., the development of safety-net institutions to improve access to primary care, interventions for improving coordination of care and discharge planning, involvement of patients and caregivers in discharge). In addition, French hospitals are under pressure to make cost savings and, reducing LOS is strongly advocated. Although the average LOS has decreased substantially over the years in France, there is still pressure to pursue reductions. Future studies should thus explore the consequences of this health policy, and in particular its impact on rehospitalization and more generally on quality of care.

Despite this low unplanned rehospitalization rate, our findings also indicate that the possibility of unplanned rehospitalization remains high for patients with certain characteristics, suggesting it could be beneficial to target interventions for patients at the greatest risk. The association between older age and rehospitalization had already been found in previous studies [4,15,16,44,45], confirming the frailty of the elderly at discharge and the need to develop specific care transition interventions for them (*e*.*g*., comprehensive inpatient geriatric health care assessment followed by ongoing multidisciplinary support after discharge, plus involvement of the patients and their caregivers) [9,46,47]. Furthermore, men were more often rehospitalized than women. Gender differences in the use of ambulatory care, higher in women compared to men, has previously been described [26,48,49], and is a complex phenomenon involving differences in illness severity along with social and cultural specificities which should be further explored in France to provide equal care for both men and women. State-funded medical assistance was associated with higher rehospitalization. This finding is not surprising, as recent works have already reported that undocumented migrant patients had high levels of chronic illness and low consultation rates to physicians in France [50,51]. In addition, this state medical assistance is underused, accessible to only 10.2% of undocumented migrant patients [52]. It should probably be improved and incorporated with France’s free universal health care, despite the current unfavorable political climate, in order to improve access to healthcare for migrants and reduce their level of rehospitalization. As in previous studies, several categories of disease, illness severity, and higher Charlson comorbidity indices were associated with higher readmission rates [42,53,54]. The strongest association concerned newborns and perinatal diseases, toxicology, pulmonology, psychiatry, chronic pain and palliative care, and digestive diseases. These particular conditions must therefore be prioritized to reduce rehospitalization.

Prior hospitalizations, especially via emergency departments, long LOS, and hospitalizations conducted via emergency departments were important predictors of unplanned rehospitalization. These factors may account for the total burden of illness, functional status, and social environment [19,34,55,56], causing more frequent rehospitalization. Importantly, short LOS was also associated with rehospitalization, confirming that current financial injunctions, particularly those that incite the development of ambulatory care, should be accompanied by appropriate reorganization of care processes to avoid detrimental effects on quality of care [57]. Lastly, return to home was associated with more rehospitalizations, thus confirming the necessity for clinicians to better prepare discharges, check the availability of home-based services, and carefully plan the transition of care. This is clearly a weak point of the FHS [16,43].

Lastly, our final aim was to propose a predictive rehospitalization risk score and, to our knowledge, this was the first unplanned 30-day rehospitalization risk model to use an understanding set of factors sourced directly from the French hospital medico-administrative database. This score is easy to use, accurate in predicting the risk of rehospitalization via emergency departments, it already presents higher discriminative properties than the LACE score (c statistics = 0.74 vs. 0.66 for the LACE index), despite being recommended by the French Health Authority. The French policy is mainly based on the publication of guidelines by the French Health Authority (HAS) [2], which recommends identifying patients at risk using the LACE index [34] or 8Ps risk assessment tools used in the BOOST (*Better Outcomes for Older adults through Safe Transitions)* program involving 11 hospitals in the USA [58]. Despite the interest of these two instruments, they have not yet been rigorously validated in France. The LACE index presented poor discriminative ability starting from its construction [18,34] and the authors themselves later improved it (AUC for 30-day urgent readmission between 0.743 and 0.753, depending on the inclusion of case mix groups) by adding other covariates closer than those used in our model but validated on the Ontario administrative database [35]. The 8Ps check list issued from the BOOST could be cumbersome in routine practice if performed for every hospitalized patient since it requires physicians to identify and address each of these factors then propose an appropriate intervention [59]. An important advantage of our predictive rehospitalization risk score is that it does not require additional completion by physicians to that already required for the PMSI; consequently, even if analyzing such a medico- administrative database may require computational aid, it is important to use the data already available and not to increase doctors' medico-administrative work burdens [60].

### Perspectives and limitations

Our findings must be interpreted in the context of our study’s limitations.

Despite the large overall sample size of this multi-center study, our findings may not be applicable to all French hospitals, particularly concerning general hospitals whose patients offer potentially different characteristics from those of university hospitals. In addition, the four university hospitals included in our study were located in only one geographical area, even though social and healthcare geographical characteristics (e.g., poverty, density of physicians, number of beds, and private hospitals) are known to influence to the risk of rehospitalization [4,20,40,61]. Future studies should thus be conducted in different categories of hospitals and several geographical areas to confirm the properties and interest of our predictive risk-score. An external validation in addition to the internal validation performed in this study will guaranty the generalizability of this score.

Our model does not take into account deaths outside the hospital since we do not dispose of this information in our database. Other studies with available data on outpatient events are needed to investigate to what extent this could impact our predictive risk score using a competing risk model as an example.

In this study, we excluded from the analyses the ambulatory surgery. However, this specific topic should be better studied in the French context, strongly marked by pressures for reducing length of stay and consequently cost of care.

Even if our predictive risk-score led to better AUC score than other scores already performed, other and more advanced methods like machine learning are advocated to investigate whether they can give better predictive power than our score derived from the logistic regression. The advantages of these methods is their ability to use more information that cannot be used with classical statistical methods such as logistic regressions, and in particular textual information in electronic patient records. Future works should use all the data relevant in hospital databases and these new methods to improve the level of prediction.

A substantial amount of data (*e*.*g*., polypharmacy, socio-economic status, and self-reported functional status) has been reported as predictive of rehospitalization [9,20,61–63], though it is not currently available in the French PMSI database. Future works should explore how to systematically collect this data in the other available databases in hospitals (*e*.*g*., electronic medical records) and if such data could improve the accuracy of our predictive rehospitalization risk score based on the PMSI database.

Another perspective of our research would be to disentangle rehospitalizations for a previously known affection from those for other and unknown affection, and thus to better precise the part of avoidable hospitalizations. This difference has been explored by Halfon et al. [41] who developed the SQLape [64] algorithm to identify avoidable hospitalizations based on specific diagnosis and specific interventions.

Lastly, the majority of predictive risk scores are based on data at discharge while they should ideally give information early enough during the hospitalization to trigger transitional care intervention [20]. While these instruments based on discharge data have been proven to lead to greater models with better performance [20,65] than models based solely on admission data, some authors have argued that this improvement was limited [65]. To address this duality, an interesting perspective study would be to implement real-time predictive rehospitalization risk scores during hospitalization, updated for all new available data, then to propose early alerts for high risk of rehospitalization. Recent works reported that machine learning methods can be used in real-time predictions using routinely collected clinical data exclusively, without the need for any manual processing [66].

## Conclusion

The 3.5% unplanned rehospitalization rate was substantially lower in our study than that of studies performed in other countries, suggesting that universal insurance coverage may be a key factor for controlling rehospitalization. Despite this low unplanned rehospitalization rate, our findings likewise indicate that the possibility of unplanned rehospitalization remains high for patients with certain characteristics, suggesting the interest of proposing targeted interventions for patients at the greatest risk. We also developed an easy-to-use predictive rehospitalization risk-score of unplanned 30-day all-cause rehospitalizations with satisfactory discriminant properties. Future works should, however, explore if other data available in electronic medical records and other databases could improve the accuracy of our predictive rehospitalization risk score based on medico-administrative data. Finally, further research is required to determine whether such quantification risk modifies *in fine* in real-life patient care and outcomes.

## Supporting information

S1 Table Charlson details of comorbidities. (DOCX)

## Abbreviations

AME: French state-funded medical assistance (Aide Médicale d’Etat)
AUC: Area Under the Curve
CMU: free universal health care (Couverture Maladie Universelle)
APHM: Assistance Publique–Hôpitaux de Marseille
LOS: length of stay
PMSI: Programme de Médicalisation des Systèmes d’Information
ROC: Receiver Operation Characteristics

## References

[1] Boutwell AE, Johnson MB, Rutherford P, Watson SR, Vecchioni N, Auerbach BS, et al An early look at a four-state initiative to reduce avoidable hospital readmissions. Health Aff Proj Hope. 2011 7;30(7):1272–80. 10.1377/hlthaff.2011.0111 21734200

[2] HAS- Haute Autorité de Santé. Note méthodologique et de synthèse documentaire «Sortie d’hospitalisation supérieure à 24 heures–Établissement d’une check-list». In. Available from: http://www.has-sante.fr/portail/upload/docs/application/pdf/2015-05/note_documentaire_check-list_sortie_hospitalisation_web.pdf

[3] Jencks SF, Williams MV, Coleman EA. Rehospitalizations among patients in the Medicare fee-for-service program. N Engl J Med. 2009 4 2;360(14):1418–28. 10.1056/NEJMsa0803563 19339721

[4] Gusmano M, Rodwin V, Weisz D, Cottenet J, Quantin C. Comparison of rehospitalization rates in France and the United States. J Health Serv Res Policy. 2015 1 1;20(1):18–25. 10.1177/1355819614551849 25256091

[5] Friedman B, Basu J. The rate and cost of hospital readmissions for preventable conditions. Med Care Res Rev MCRR. 2004 6;61(2):225–40. 10.1177/1077558704263799 15155053

[6] Ashton CM, Kuykendall DH, Johnson ML, Wray NP, Wu L. The association between the quality of inpatient care and early readmission. Ann Intern Med. 1995 3 15;122(6):415–21. 7856989 10.7326/0003-4819-122-6-199503150-00003

[7] Balla U, Malnick S, Schattner A. Early readmissions to the department of medicine as a screening tool for monitoring quality of care problems. Medicine (Baltimore). 2008 9;87(5):294–300. 18794712 10.1097/MD.0b013e3181886f93

[8] François P, Bertrand D, Beden C, Fauconnier J, Olive F. [Early readmission as an indicator of hospital quality of care]. Rev Epidemiol Sante Publique. 2001 4;49(2):183–92. 11319485

[9] Coleman EA, Parry C, Chalmers S, Min S-J. The care transitions intervention: results of a randomized controlled trial. Arch Intern Med. 2006 9 25;166(17):1822–8. 10.1001/archinte.166.17.1822 17000937

[10] Leppin AL, Gionfriddo MR, Kessler M, Brito JP, Mair FS, Gallacher K, et al Preventing 30-day hospital readmissions: a systematic review and meta-analysis of randomized trials. JAMA Intern Med. 2014 7;174(7):1095–107. 10.1001/jamainternmed.2014.1608 24820131 PMC4249925

[11] Atete-Leblanc R, Bréchat P-H, Morel O, Thoury A, Frati A, Barranger E. [Precarious parturients and readmission: pilot study at the Lariboisière-Fernand-Widal Hospital Group in Paris]. Gynecol Obstet Fertil. 2012 12;40(12):753–8. 10.1016/j.gyobfe.2011.08.020 22503489

[12] Dely C, Sellier P, Dozol A, Segouin C, Moret L, Lombrail P. [Preventable readmissions of “community-acquired pneumonia”: Usefulness and reliability of an indicator of the quality of care of patients’ care pathways]. Presse Medicale Paris Fr 1983 2012 1;41(1):e1–9. 10.1016/j.lpm.2011.06.007 21802247

[13] Caughey GE, Pratt NL, Barratt JD, Shakib S, Kemp-Casey AR, Roughead EE. Understanding 30-day re-admission after hospitalisation of older patients for diabetes: identifying those at greatest risk. Med J Aust. 2017 3 6;206(4):170–5. 28253467 10.5694/mja16.00671

[14] Lam C, Chen P-L, Kang J-H, Cheng K-F, Chen R-J, Hung K-S. Risk factors for 14-day rehospitalization following trauma with new traumatic spinal cord injury diagnosis: A 10-year nationwide study in Taiwan. PloS One. 2017;12(9):e0184253 10.1371/journal.pone.0184253 28863195 PMC5581159

[15] Crane SJ, Tung EE, Hanson GJ, Cha S, Chaudhry R, Takahashi PY. Use of an electronic administrative database to identify older community dwelling adults at high-risk for hospitalization or emergency department visits: the elders risk assessment index. BMC Health Serv Res. 2010 12 13;10:338 10.1186/1472-6963-10-338 21144042 PMC3019201

[16] Mesteig M, Helbostad JL, Sletvold O, Røsstad T, Saltvedt I. Unwanted incidents during transition of geriatric patients from hospital to home: a prospective observational study. BMC Health Serv Res. 2010 1 4;10:1 10.1186/1472-6963-10-1 20044945 PMC2827472

[17] Davidoff F, Batalden P, Stevens D, Ogrinc G, Mooney S, for the SQUIRE Development Group. Publication Guidelines for Improvement Studies in Health Care: Evolution of the SQUIRE Project. Ann Intern Med. 2008 11 4;149(9):670 18981488 10.7326/0003-4819-149-9-200811040-00009

[18] Garrison GM, Robelia PM, Pecina JL, Dawson NL. Comparing performance of 30-day readmission risk classifiers among hospitalized primary care patients. J Eval Clin Pract. 2016 10 3; 10.1111/jep.12656 27696638

[19] Donzé J, Aujesky D, Williams D, Schnipper JL. Potentially avoidable 30-day hospital readmissions in medical patients: derivation and validation of a prediction model. JAMA Intern Med. 2013 4 22;173(8):632–8. 10.1001/jamainternmed.2013.3023 23529115

[20] Kansagara D, Englander H, Salanitro A, Kagen D, Theobald C, Freeman M, et al Risk prediction models for hospital readmission: a systematic review. JAMA. 2011 10 19;306(15):1688–98. 10.1001/jama.2011.1515 22009101 PMC3603349

[21] Strom JB, Kramer DB, Wang Y, Shen C, Wasfy JH, Landon BE, et al Short-term rehospitalization across the spectrum of age and insurance types in the United States. PloS One. 2017;12(7):e0180767 10.1371/journal.pone.0180767 28700736 PMC5507267

[22] Horwitz L. The insurance-readmission paradox: why increasing insurance coverage may not reduce hospital-level readmission rates. J Hosp Med. 2014 11;9(11):743–4. 10.1002/jhm.2271 25303367

[23] Lasser KE, Hanchate AD, McCormick D, Manze MG, Chu C, Kressin NR. The effect of Massachusetts health reform on 30 day hospital readmissions: retrospective analysis of hospital episode statistics. BMJ. 2014 3 31;348:g2329 10.1136/bmj.g2329 24687184 PMC3970763

[24] Chen C, Scheffler G, Chandra A. Readmission penalties and health insurance expansions: A dispatch from Massachusetts. J Hosp Med. 2014 11 1;9(11):681–7. 10.1002/jhm.2213 24945696

[25] Schiltz NK, Finkelstein Rosenthal B, Crowley MA, Koroukian SM, Nevar A, Meropol SB, et al Rehospitalization during the first year of life by insurance status. Clin Pediatr (Phila). 2014 8;53(9):845–53. 24899633 10.1177/0009922814536924 PMC4412744

[26] Gusmano MK, Rodwin VG, Weisz D. A new way to compare health systems: avoidable hospital conditions in Manhattan and Paris. Health Aff Proj Hope. 2006 4;25(2):510–20. 10.1377/hlthaff.25.2.510 16522605

[27] Rodwin VG. The health care system under French national health insurance: lessons for health reform in the United States. Am J Public Health. 2003 1;93(1):31–7. 12511380 10.2105/ajph.93.1.31 PMC1447687

[28] Rodwin VG, Le Pen C. Health care reform in France—the birth of state-led managed care. N Engl J Med. 2004 11 25;351(22):2259–62. 10.1056/NEJMp048210 15564541

[29] Direction générale de l’offre de soins (DGOS) Ministère des Solidarités et de la Santé. Les indicateurs de ré-hospitalisation et de coordination [Internet]. Available from: http://solidarites-sante.gouv.fr/soins-et-maladies/qualite-des-soins-et-pratiques/qualite/les-indicateurs/article/re-hospitalisation-coordination

[30] Bottle A, Aylin P, Majeed A. Identifying patients at high risk of emergency hospital admissions: a logistic regression analysis. J R Soc Med. 2006 8;99(8):406–14. 10.1258/jrsm.99.8.406 16893941 PMC1533524

[31] World Health Organization. ICD-10 Version:2010 [Internet]. [cited 2017 Nov 10]. Available from: http://apps.who.int/classifications/icd10/browse/2010/en

[32] Quan H, Sundararajan V, Halfon P, Fong A, Burnand B, Luthi J-C, et al Coding algorithms for defining comorbidities in ICD-9-CM and ICD-10 administrative data. Med Care. 2005 11;43(11):1130–9. 16224307 10.1097/01.mlr.0000182534.19832.83

[33] ATIH. Manuel des GHL- version 11d [Internet]. [cited 2018 Nov 19]. Available from: https://www.atih.sante.fr/manuel-des-ghm-version-11d

[34] van Walraven C, Dhalla IA, Bell C, Etchells E, Stiell IG, Zarnke K, et al Derivation and validation of an index to predict early death or unplanned readmission after discharge from hospital to the community. CMAJ Can Med Assoc J J Assoc Medicale Can. 2010 4 6;182(6):551–7. 10.1503/cmaj.091117 PMC2845681 20194559

[35] van Walraven C, Wong J, Forster AJ. LACE+ index: extension of a validated index to predict early death or urgent readmission after hospital discharge using administrative data. Open Med Peer-Rev Indep Open-Access J. 2012;6(3):e80–90. PMC3659212 23696773

[36] Moons KGM, Harrell FE, Steyerberg EW. Should scoring rules be based on odds ratios or regression coefficients? J Clin Epidemiol. 2002 10;55(10):1054–5. 12464384 10.1016/s0895-4356(02)00453-5

[37] All. Data Mining et statistique décisionnelle—TUFFERY Stéphane [Internet]. Technip. [cited 2017 Mar 15]. Available from: http://www.editionstechnip.com/fr/catalogue-detail/1005/data-mining-et-statistique-decisionnelle.html

[38] Analyzing Receiver Operating Characteristic Curves with SAS [Internet]. [cited 2017 Mar 15]. Available from: https://www.sas.com/store/prodBK_60610_en.html

[39] Gonen M. Analyzing Receiver Operating Characteristic Curves with SAS [Internet]. SAS Insitute. 2007. Available from: https://www.sas.com/store/books/categories/usage-and-reference/analyzing-receiver-operating-characteristic-curves-with-sas-/prodBK_60610_en.html

[40] Hernandez AF, Greiner MA, Fonarow GC, Hammill BG, Heidenreich PA, Yancy CW, et al Relationship between early physician follow-up and 30-day readmission among Medicare beneficiaries hospitalized for heart failure. JAMA. 2010 5 5;303(17):1716–22. 10.1001/jama.2010.533 20442387

[41] Halfon P, Eggli Y, Prêtre-Rohrbach I, Meylan D, Marazzi A, Burnand B. Validation of the potentially avoidable hospital readmission rate as a routine indicator of the quality of hospital care. Med Care. 2006 11;44(11):972–81. 10.1097/01.mlr.0000228002.43688.c2 17063128

[42] Wong ELY, Cheung AWL, Leung MCM, Yam CHK, Chan FWK, Wong FYY, et al Unplanned readmission rates, length of hospital stay, mortality, and medical costs of ten common medical conditions: a retrospective analysis of Hong Kong hospital data. BMC Health Serv Res. 2011 6 17;11:149 10.1186/1472-6963-11-149 21679471 PMC3146405

[43] François P, Boussat B, Fourny M, Seigneurin A. [Quality of service provided by a university hospital: general practitioners’ opinion]. Sante Publique Vandoeuvre—Nancy Fr. 2014 4;26(2):189–97. 25108960

[44] Ben-Chetrit E, Chen-Shuali C, Zimran E, Munter G, Nesher G. A simplified scoring tool for prediction of readmission in elderly patients hospitalized in internal medicine departments. Isr Med Assoc J IMAJ. 2012 12;14(12):752–6. 23393714

[45] Aljishi M, Parekh K. Risk factors for general medicine readmissions and association with mortality. N Z Med J. 2014 5 23;127(1394):42–50. 24929570

[46] Conroy SP, Stevens T, Parker SG, Gladman JRF. A systematic review of comprehensive geriatric assessment to improve outcomes for frail older people being rapidly discharged from acute hospital: “interface geriatrics.” Age Ageing. 2011 7;40(4):436–43. 10.1093/ageing/afr060 21616954

[47] Conroy S, Dowsing T. What should we do about hospital readmissions? Age Ageing. 2012 11;41(6):702–4. 10.1093/ageing/afs154 23045361

[48] Bertakis KD, Azari R, Helms LJ, Callahan EJ, Robbins JA. Gender differences in the utilization of health care services. J Fam Pract. 2000 2;49(2):147–52. 10718692

[49] Muller C. Health Care and Gender Russell Sage Foundation; 1990. 273 p.

[50] Dourgnon P. et al Le recours aux soins de ville des immigrés en France Questions d’économie de la santé, n° 146, Paris, IRDES 2009;

[51] Berchet C, Jusot F. Inégalités de santé liées à l’immigration et capital social: une analyse en décomposition. Économie PubliquePublic Econ. 2012 11 15;(24–25):73–100.

[52] André J-M, Azzedine F. Access to healthcare for undocumented migrants in France: a critical examination of State Medical Assistance. Public Health Rev. 2016 8 3;37:5 10.1186/s40985-016-0017-4 29450047 PMC5809954

[53] Jou H-J, Siao R-Y, Tsai Y-S, Chen Y-T, Li C-Y, Chen C-C. Postdischarge rehospitalization and in-hospital mortality among Taiwanese women with hip fracture. Taiwan J Obstet Gynecol. 2014 3;53(1):43–7. 10.1016/j.tjog.2012.04.042 24767645

[54] Kates SL, Behrend C, Mendelson DA, Cram P, Friedman SM. Hospital readmission after hip fracture. Arch Orthop Trauma Surg. 2015 3;135(3):329–37. 10.1007/s00402-014-2141-2 25550095

[55] Hasan O, Meltzer DO, Shaykevich SA, Bell CM, Kaboli PJ, Auerbach AD, et al Hospital readmission in general medicine patients: a prediction model. J Gen Intern Med. 2010 3;25(3):211–9. 10.1007/s11606-009-1196-1 20013068 PMC2839332

[56] Billings J, Dixon J, Mijanovich T, Wennberg D. Case finding for patients at risk of readmission to hospital: development of algorithm to identify high risk patients. BMJ. 2006 8 12;333(7563):327 10.1136/bmj.38870.657917.AE 16815882 PMC1539047

[57] Kossovsky MP, Sarasin FP, Chopard P, Louis-Simonet M, Sigaud P, Perneger TV, et al Relationship between hospital length of stay and quality of care in patients with congestive heart failure. BMJ Qual Saf. 2002 9 1;11(3):219–23. 10.1136/qhc.11.3.219 PMC1743633 12486984

[58] Hansen LO, Greenwald JL, Budnitz T, Howell E, Halasyamani L, Maynard G, et al Project BOOST: effectiveness of a multihospital effort to reduce rehospitalization. J Hosp Med. 2013 8;8(8):421–7. 10.1002/jhm.2054 23873709

[59] Risk Assessment Tool: the 8Ps [Internet]. [cited 2017 Apr 26]. Available from: http://www.hospitalmedicine.org/Web/Quality_Innovation/Implementation_Toolkits/Project_BOOST/Web/Quality___Innovation/Implementation_Toolkit/Boost/BOOST_Intervention/Tools/Risk_Assessment.aspx

[60] Woolhandler S, Himmelstein DU. Administrative work consumes one-sixth of U.S. physicians’ working hours and lowers their career satisfaction. Int J Health Serv Plan Adm Eval. 2014;44(4):635–42. 10.2190/HS.44.4.a 25626223

[61] Kripalani S, Jackson AT, Schnipper JL, Coleman EA. Promoting effective transitions of care at hospital discharge: a review of key issues for hospitalists. J Hosp Med. 2007 9;2(5):314–23. 10.1002/jhm.228 17935242

[62] Morrissey EF, McElnay JC, Scott M, McConnell BJ. Influence of Drugs, Demographics and Medical History on Hospital Readmission of Elderly Patients: A Predictive Model. Clin Drug Investig. 2003;23(2):119–28.

[63] Amarasingham R, Moore BJ, Tabak YP, Drazner MH, Clark CA, Zhang S, et al An automated model to identify heart failure patients at risk for 30-day readmission or death using electronic medical record data. Med Care. 2010 11;48(11):981–8. 10.1097/MLR.0b013e3181ef60d9 20940649

[64] SQLape [Internet]. [cited 2018 Nov 19]. Available from: http://www.sqlape.com/AR_ALGORITHM.htm

[65] Nguyen OK, Makam AN, Clark C, Zhang S, Xie B, Velasco F, et al Predicting all-cause readmissions using electronic health record data from the entire hospitalization: Model development and comparison. J Hosp Med. 2016 7;11(7):473–80. 10.1002/jhm.2568 26929062 PMC5365027

[66] Meyer A, Zverinski D, Pfahringer B, Kempfert J, Kuehne T, Sündermann SH, et al Machine learning for real-time prediction of complications in critical care: a retrospective study. Lancet Respir Med. 2018 9 28 10.1016/S2213-2600(18)30300-X 30274956

