# Supplementary material for: Predictive risk score for unplanned 30-day rehospitalizations in the French universal health care system based on a medico-administrative database
Source: PLoS One. 2019 Mar 12;14(3):e0210714. doi: 10.1371/journal.pone.0210714 (PMC6414180; doi:10.1371/journal.pone.0210714)
Supplement: S1 Table — (DOCX) [file pone.0210714.s001.docx]

Supplementary Table 1: Charlson details of comorbidities

| **Charlson Detail of comorbidities** | **30 days re-hospitalization rates** | | **p-value** | **All (n=118 650)** | |
| --- | --- | --- | --- | --- | --- |
|  | **N (4 127)** | **%** | **<0,0001** | **N** | **% of all** |
| **Myocardial infarction** |  |  | **<0,0001** |  |  |
| No | 3 858 | 3,4 |  | 113 544 | 95,7 |
| Yes | 269 | 5,3 |  | 5 106 | 4,3 |
| **Congestive Heart Failure** |  |  | **<0,0001** |  |  |
| No | 3 821 | 3,4 |  | 113 880 | 96,0 |
| Yes | 306 | 6,4 |  | 4 770 | 4,0 |
| **Peripheral vascular disease** |  |  | **0,25** |  |  |
| No | 4 039 | 3,5 |  | 116 413 | 98,1 |
| Yes | 88 | 3,9 |  | 2 237 | 1,9 |
| **Cerebrovascular disease** |  |  | **0,01** |  |  |
| No | 4 016 | 3,5 |  | 116 128 | 97,9 |
| Yes | 111 | 4,4 |  | 2 522 | 2,1 |
| **Dementia** |  |  | **<0,0001** |  |  |
| No | 3 916 | 3,4 |  | 116 345 | 98,1 |
| Yes | 211 | 9,2 |  | 2 305 | 1,9 |
| **Chronic pulmonary disease** |  |  | **<0,0001** |  |  |
| No | 3 941 | 3,4 |  | 115 459 | 97,3 |
| Yes | 186 | 5,8 |  | 3 191 | 2,7 |
| **Rheumatologic disease** |  |  | **0,74** |  |  |
| No | 4 104 | 3,5 |  | 117 942 | 99,4 |
| Yes | 23 | 3,3 |  | 708 | 0,6 |
| **Peptic ulcer disease** |  |  | **0,009** |  |  |
| No | 4 110 | 3,5 |  | 118 384 | 99,8 |
| Yes | 17 | 6,4 |  | 266 | 0,2 |
| **Mild liver disease** |  |  | **<0,0001** |  |  |
| No | 4 006 | 3,4 |  | 116 318 | 98,0 |
| Yes | 121 | 5,2 |  | 2 332 | 2,0 |
| **Diabetes without chronic complications** |  |  | **<0,0001** |  |  |
| No | 3 829 | 3,4 |  | 111 997 | 94,4 |
| Yes | 298 | 4,5 |  | 6 653 | 5,6 |
| **Diabetes with chronic complications** |  |  | **0,006** |  |  |
| No | 3 975 | 3,5 |  | 115 128 | 97,0 |
| Yes | 152 | 4,3 |  | 3 522 | 3,0 |
| **Hemiplegia of paraplegia** |  |  | **0,04** |  |  |
| No | 4 013 | 3,5 |  | 115 920 | 97,7 |
| Yes | 114 | 4,2 |  | 2 730 | 2,3 |
| **Renal disease** |  |  |  |  |  |
| No | 3 938 | 3,5 | **0,02** | 114 058 | 96,1 |
| Yes | 189 | 4,1 |  | 4 592 | 3,9 |
| **Any malignancy including leukemia and lymphoma** | |  | **<0,0001** |  |  |
| **No** | 3 803 | 3,4 |  | 112 312 | 94,7 |
| Yes | 324 | 5,1 |  | 6 338 | 5,3 |
| **Moderate or severe liver disease** | 4 075 | 3,5 | **<0,0001** | 118 093 | 99,5 |
| No |  |  |  |  |  |
| Yes | 52 | 9,3 |  | 557 | 0,5 |
| **Metastatic Solid Tumor** |  |  | **<0,0001** |  |  |
| No | 3 933 | 3,4 |  | 115 198 | 97,1 |
| Yes | 194 | 5,6 |  | 3 452 | 2,9 |
| **HIV** |  |  | **0,71** |  |  |
| No | 4 114 | 3,5 |  | 118 236 | 99,7 |
| Yes | 13 | 3,1 |  | 414 | 0,4 |
